# Ohio Medical and Surg. Journal

**Published:** 1853-10

**Authors:** 

**Affiliations:** Prof. of Surgery in the Starling Medical College, at Columbus, Ohio


The Ohio Medical and Surgical Journal.—*Edited by R. L. Howard, M. D., Prof. of Surgery in the Starling Medical College, at Columbus, Ohio.*—This valuable Western periodical come to us a few days since, laden, as usual, with its choice offerings. No journal of the country has sustained itself better, in point of utility and usefulness, than this bi-monthly periodical. The profession of Ohio have good reason to congratulate themselves that, in their midst, they have so efficient an organ and one conducted with so much ability. We hope it is liberally and generously sustained, and that it is to be found in the library of every respectable physician in the State. Nothing so much exalts the profession of a State, in the estimation of the general medical public, as to know that they hold up the hands of those engaged in elevating, higher and higher, the standard of the profession, and in the dissemination of truth and light throughout the entire mass of the medical men of the State. It shows conclusively that the medical mind is in the ascendant, and that there is a spirit of enquiry abroad, extending every where, and seizing with avidity, upon every means of advancement and improvement.

